# HIF1α-dependent hypoxia response in myeloid cells requires IRE1α

**DOI:** 10.1186/s12974-023-02793-y

**Published:** 2023-06-21

**Authors:** Gaëlle Mawambo, Malika Oubaha, Yusuke Ichiyama, Guillaume Blot, Sergio Crespo-Garcia, Agnieszka Dejda, François Binet, Roberto Diaz-Marin, Christina Sawchyn, Mikhail Sergeev, Rachel Juneau, Randal J. Kaufman, El Bachir Affar, Frédérick A. Mallette, Ariel M. Wilson, Przemyslaw Sapieha

**Affiliations:** 1 Present Address: Department of Biochemistry, Maisonneuve-Rosemont Hospital Research Centre, Université de Montréal, 5415 De L’Assomption Boulevard, Montréal, QC H1T 2M4 Canada; 2 Department of Ophthalmology, Maisonneuve-Rosemont Hospital Research Centre, Université de Montréal, Montréal, QC H1T 2M4 Canada; 3 Départment de Sciences Biologiques, Université du Québec À Montréal (UQAM), Montréal, QC H2X 1L4 Canada; 4 Degenerative Diseases Program, Sanford Burnham Prebys Medical Discovery Institute, 10901 N. Torrey Pines Rd, La Jolla, CA 92037 USA; 5 School of Optometry, University of Montreal, Montreal, QC H3T1P1 Canada; 6 Department of Medicine, University of Montreal, Montreal, Canada

**Keywords:** HIF1α, Retina, Angiogenesis, Inflammation, IRE1α, Myeloid, Mononuclear phagocytes, Microglia, Hypoxia, ER stress

## Abstract

Cellular adaptation to low oxygen tension triggers primitive pathways that ensure proper cell function. Conditions of hypoxia and low glucose are characteristic of injured tissues and hence successive waves of inflammatory cells must be suited to function under low oxygen tension and metabolic stress. While Hypoxia-Inducible Factor (HIF)-1α has been shown to be essential for the inflammatory response of myeloid cells by regulating the metabolic switch to glycolysis, less is known about how HIF1α is triggered in inflammation. Here, we demonstrate that cells of the innate immune system require activity of the inositol-requiring enzyme 1α (IRE1α/XBP1) axis in order to initiate HIF1α-dependent production of cytokines such as IL1β, IL6 and VEGF-A. Knockout of either HIF1α or IRE1α in myeloid cells ameliorates vascular phenotypes in a model of retinal pathological angiogenesis driven by sterile inflammation. Thus, pathways associated with ER stress, in partnership with HIF1α, may co-regulate immune adaptation to low oxygen.

**Supplementary Information:**

The online version contains supplementary material available at 10.1186/s12974-023-02793-y.

## Introduction

Cells of myeloid lineage are highly motile and dynamic early responders to invading pathogens and non-microbial tissue damage [1–3]. They are called to operate under conditions of environmental stress such as extreme hypoxia found in infected tissues, tumors and ischemic CNS [4]. As myeloid cells enter sites of distressed tissues, they engage adaptive responses to cope with the microenvironment that they are called to defend or repair [5, 6]. Tissue injury provokes a series of biochemical events that reduce oxygen tension and glucose levels in damaged cells [7]. Hence, as immune cells hone in on injured tissue, they must be suited to function under ischemic and metabolic stress.

When facing oxygen deprivation, cells activate a set of adaptive mechanisms. A crucial oxygen-sensing effector is the transcription factor Hypoxia-Inducible Factor 1 (HIF1), a heterodimeric protein containing an oxygen-sensitive α subunit and a nuclear localized stable β subunit [8–13]. In well-oxygenated environments, HIF1α is hydroxylated by prolyl hydroxylase domain proteins and targeted for proteasomal degradation by E3 ubiquitin ligase through binding to the von Hippel–Lindau tumor suppressor protein [8–11]. Under conditions of hypoxic stress, HIF1α is stabilized and participates in regulating adaptive processes such as angiogenesis and inflammation.

Beyond adjustment to oxygen levels, myeloid cells engage HIF1α during the inflammatory response to aid in tissue infiltration and activation through regulation of glycolytic capacity [14, 15]. With the goal of identifying modulators of HIF1α function, we set out to elucidate contemporaneous events that are triggered when cells of myeloid origin enter hypoxic tissue. Through tandem mass spectrometry (MS/MS), we identified Glucose-Regulated Protein-78 (GRP78) as a prospective binder of HIF1α during hypoxia. GRP78 is an endoplasmic reticulum (ER) chaperone and plays important roles in the Unfolded Protein Response (UPR) [16–19].

During hypoxic stress, energetic resources are reallocated with selected transcription of mRNAs coding for proteins involved in the maintenance of cellular homeostasis [20, 21]. Part of this selective protein production is ensured through conserved pathways of the UPR in conditions of ER stress, initiated by three axis: the protein kinase RNA-like ER kinase/activating transcription factor 4 (PERK/ATF4) axis, the inositol-requiring enzyme-1α/X-box binding protein-1 (IRE1α/XBP1) axis, and the activating transcription factor 6 (ATF6) axis [17–19]. Here, we investigated the potential crosstalk of UPR pathways with HIF1α during the response of myeloid cells to hypoxic stress within the ischemic retina.

## Results

### HIF1α interacts with IRE1α during the response of myeloid cells to hypoxia

**Fig. 1:**
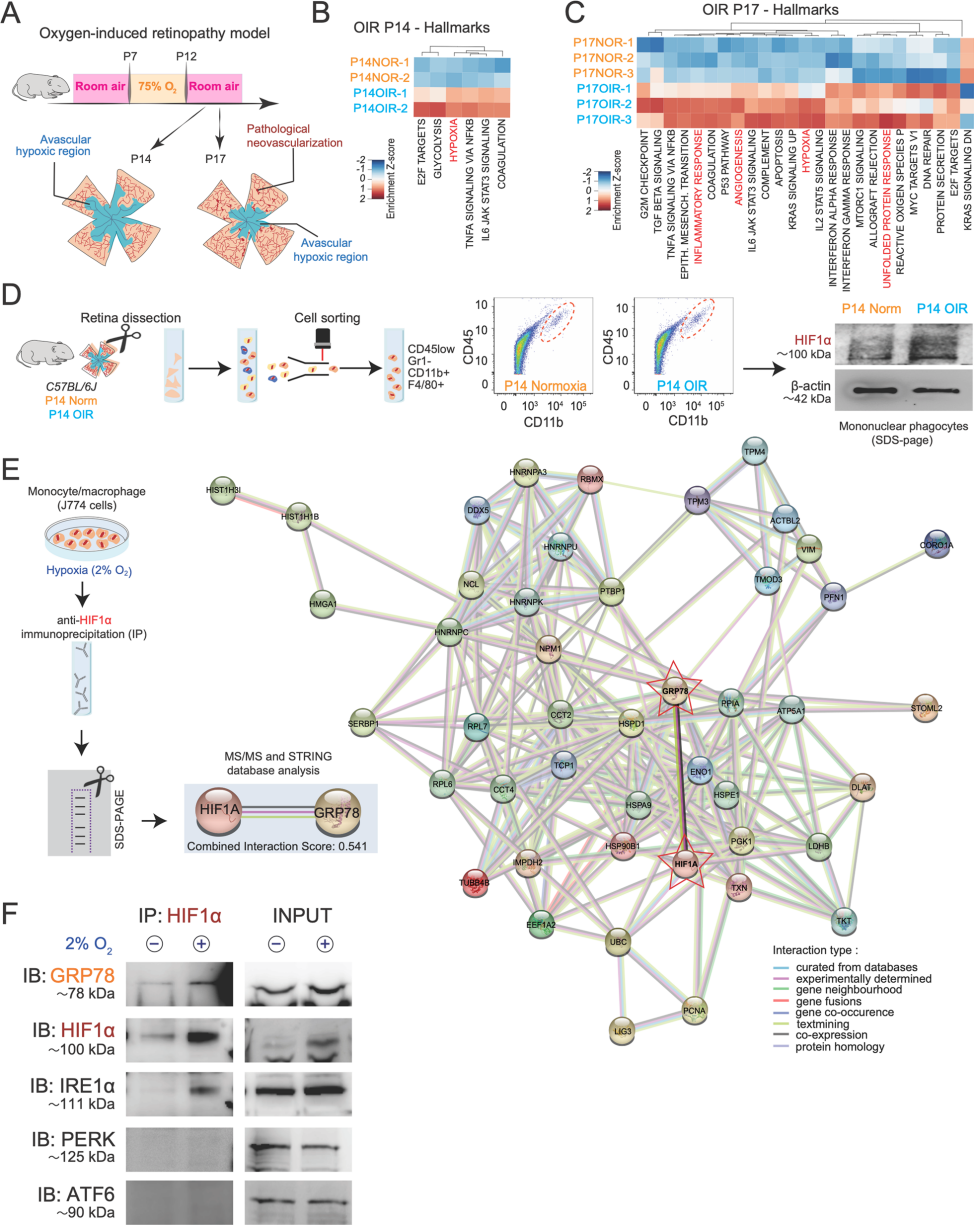
HIF1α and IRE1α interact during myeloid cell response to hypoxia. **A** Schematic representation of the OIR mouse model. **B** Heat map of gene set variation analysis (GSVA) enrichment scores of RNA-seq data from OIR and normoxic retinas at P14 and **C** P17. Pathways associated with hypoxia response are enriched at P14 when the retina is still avascular, and pathways involved in hypoxia, inflammatory responses and angiogenesis are significantly upregulated at P17 when there is maximal preretinal neovascularization; *n* = 2–3 mice per condition. For P14, *P* < 0.05 and > 0.2 logFC and for P17, p adj < 0.05 and > 0.2 logFC. **D** Immunoblot showing HIF1α stabilization in mononuclear phagocytes (CD45^low^, Gr1^−^, CD11b^+^, F4/80^+^) cell-sorted from normoxic and OIR retinas at P14. **E** STRING database representation of the protein interaction network of HIF1α immunoprecipitated from J774 macrophages under hypoxia (2% O_2_ for 8 h) and subjected to tandem mass spectrometry (MS/MS). Proteins including the unfolded protein response (UPR) such as GRP78 are highlighted in blue, and the interaction score ranked from 0 to 1 is noted below. **F** Co-immunoprecipitation of HIF1α in J774 macrophages under normoxia (21% O_2_) and hypoxia (2% O_2_) for 1 h followed by immunoblotting (IB) for UPR sensors IRE1α, PERK and ATF6 (*n* = 3 independent experiments)

To study mechanisms by which cells of myeloid origin function under hypoxic conditions, we employed the mouse model of oxygen-induced retinopathy (OIR) that is characterized by ischemic retinal tissues and deregulated angiogenesis [22]. Mouse pups were exposed to 75% oxygen from postnatal day (P) 7 to P12 to trigger vaso-obliteration, then returned to room air to initiate a second phase of pathological neovascularization that peaks at P17 (Fig. 1A). We performed bulk RNA-sequencing followed by gene set variation analysis (GSVA) on OIR retinas at P14 while the retina is revascularizing, and at P17 during peak preretinal neovascularization. As predicted, among others, we observed enrichment in genes coding for processes associated with tissue hypoxia (*P* = 0.0037) and glycolysis (*P* = 0.0403) at P14, and at P17 during maximal pathological neovascularization [23, 24], hypoxia (*P* = 0.0001), inflammation (*P* = 0.0017), angiogenesis (*P* = 5.50E−08) and UPR (*P* = 0.0016) (Fig. 1B, C; Additional file 1: Fig. S1A and Additional file 2: Table S1 and Additional file 3: Table S2). Hence, OIR models a disease state associated with hypoxia and inflammation in retinal tissue.

Pathological angiogenesis in ischemic retinopathies is driven by mononuclear phagocytes (MNPs), which include microglia, monocytes and macrophages [25–28]. We therefore proceeded to sort CD45^low^/Gr1^−^/CD11b^+^/F4/80^+^ MNPs by FACS from P14 OIR or normoxic retinas. Western blots of MNPs from P14 OIR retinas showed upregulation of HIF1α compared to normoxic controls (Fig. 1D).

To gain insight on the mechanisms by which HIF1α functions in MNPs during hypoxia, we investigated its potential binding partners. To mimic the environment MNPs encounter when entering an ischemic tissue, we subjected J774 monocyte-macrophage cells to 2% O_2_ and immunoprecipitated HIF1α followed by MS/MS. Under normoxic control conditions, we did not immunoprecipitate HIF1α. Upon hypoxia, we identified 52 proteins that precipitated with HIF1α, and inputted results into the STRING database to map out functional protein association networks [29] (Additional file 4: Table S3). Within the interactome of HIF1α, we opted to investigate GRP78 given its critical role as a chaperone involved in UPR signaling [16–19] (Fig. 1E) and hence potential to modulate production of secreted proteins such as cytokines.

UPR signaling is primarily regulated by three ER-bound transmembrane sensors, PERK, IRE1α and ATF6 [17–19]. We therefore investigated the potential binding of each UPR effector with HIF1α in hypoxic conditions. Immunoprecipitation of HIF1α from J774 cells cultured at 2% O_2_ followed by immunoblotting confirmed that GRP78 immunoprecipitated with HIF1α (Fig. 1F, Additional file 1: Fig. S1A). Interestingly, of all 3 UPR effectors, only IRE1α co-precipitated with HIF1α under hypoxic stress, while PERK and ATF6 did not (Fig. 1F). Together, these data suggest a potential collaboration between HIF1α and IRE1α in macrophages during adaptation to conditions of low oxygen tension.

### IRE1α kinase activity is required for HIF1α stabilization in myeloid response to hypoxia

**Fig. 2:**
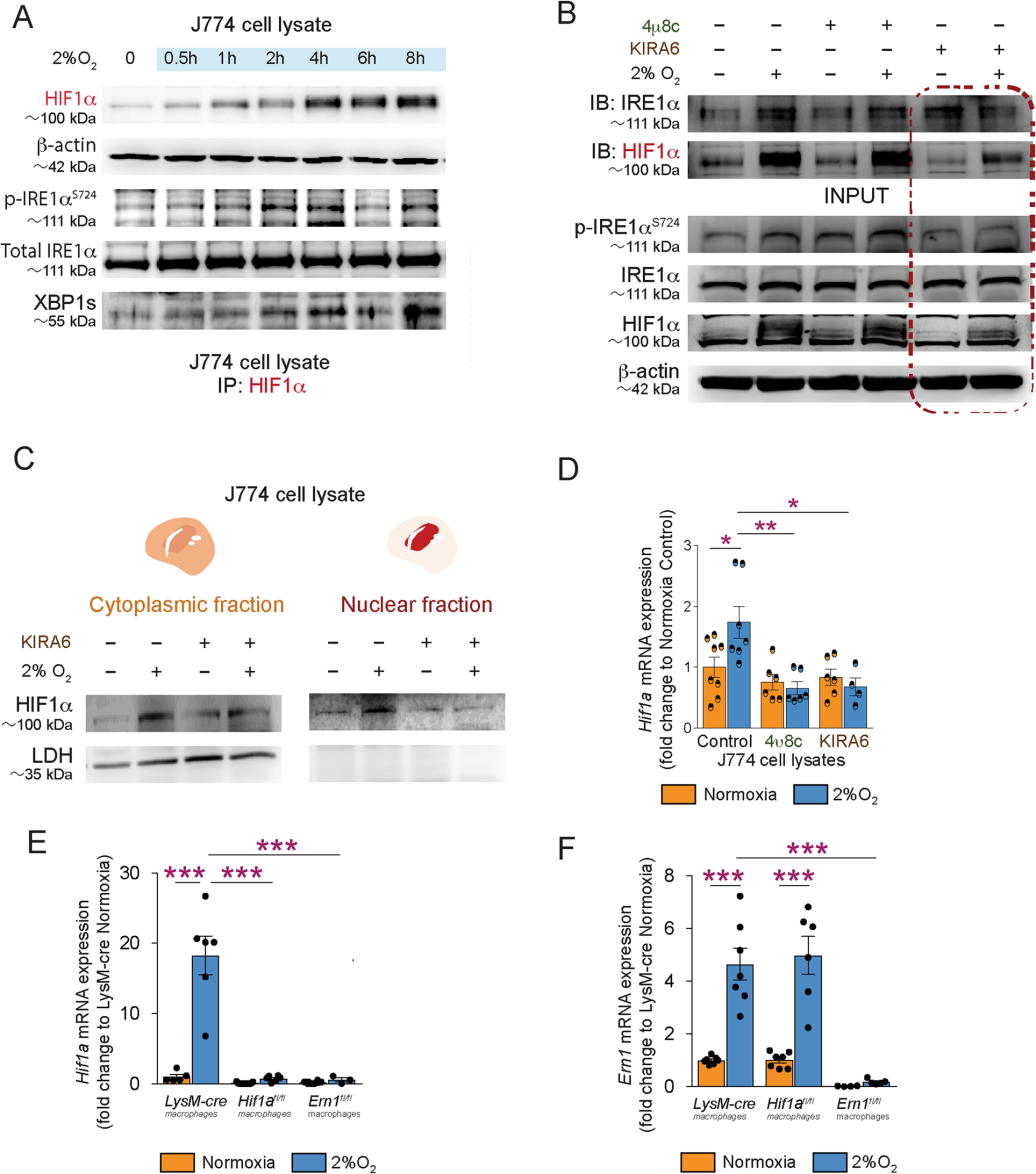
IRE1α kinase activity is required for HIF1α stabilization in macrophage response to hypoxia. **A** Immunoblot timecourse from J774 macrophage cell lysates under hypoxia probed for HIF1α stabilization, IRE1α phosphorylation and expression. (*n* = 3 independent experiments). **B** Co-immunoprecipitation of HIF1α and IRE1α in hypoxic (2% O_2_ for 1 h) J774 macrophages preincubated for 1 h with IRE1α endoribonuclease inhibitor 4µ8c (100µM) or IRE1α kinase inhibitor KIRA6 (1µM) (*n* = 3 independent experiments). Red box highlights Co-IP results upon KIRA6 treatment. **C** Immunoblots for HIF1α stabilization in cytosolic and nuclear fractions of hypoxic (2% O_2_ for 1 h) J774 cells pretreated with IRE1α kinase inhibitor KIRA6 (1µM) for 1 h. LDH was used to assess the purity of the cytosolic fraction (*n* = 3 independent experiments). **D** RT**-**qPCR analysis of *Hif1a* mRNA expression in hypoxic (2% O_2_ for 8 h) J774 cells preincubated for 1 h with IRE1α kinase inhibitor KIRA6 (1µM). *n* = 3–8 per condition, unpaired two-tailed t-test. **E** RT-qPCR analysis of *Hif1a *and **F** *Ern1* mRNA expression in LysM-*Hif1a*^−/−^ or LysM-*Ern1*^−/−^ peritoneal macrophages and their control LysM-cre*/HIF1a*^+*/*+^*/Ern1*^+*/*+^ mice under normoxic (21% O_2_) or hypoxic (2% O_2_ for 8 h) conditions. *n* = 3–12 per condition. Data expressed as mean ± S.E.M. Statistical analysis (**D**, **F**, **G**): one-way ANOVA with Bonferroni post hoc analysis; *P < 0.05, **P < 0.01, and ***P < 0.001

To study the interplay between HIF1α and IRE1α, we investigated the contribution of both cytosolic kinase and endoribonuclease functions of IRE1α. The kinase activity of IRE1α is critical for trans-autophosphorylation and activation of endoribonuclease activity. Upon activation via trans-autophosphorylation, IRE1α acquires endoribonucleolytic activity to cleave selected mRNAs and promote the splicing of XBP1 into an active transcription factor, XBP1s. XBP1s regulates the expression of genes involved in ER homeostasis [17–19]. We first assessed the dynamics of HIF1α expression and phosphorylation of IRE1α in J774 monocyte-macrophage cells under conditions of low oxygen. Consistent with their known roles as regulators of adaptation to cellular stress such as hypoxia, HIF1α expression/stabilization, IRE1α phosphorylation and generation of XBP1s were rapidly and persistently triggered through the duration of the hypoxic stimulus **(**Fig. 2A**)**.

IRE1α activity has been implicated in HIF1α signaling within endothelial cells [30]. To determine the role of the kinase and the endoribonuclease domains of IRE1α in HIF1α stability, we assessed the effects of both the IRE1α endoribonuclease inhibitor 4µ8c or kinase inhibitor KIRA6 [31] (Fig. 2B). KIRA6 dose-dependently inhibits IRE1α kinase activity and oligomerization leading to reduced XBP1 RNA cleavage and degradation of other downstream targets such as Ins2 RNA [31]. Inhibition of the IRE1α kinase domain by KIRA6 reduced hypoxia-mediated HIF1α protein stabilization (red outlined lower panel) as well as the interaction between HIF1α and IRE1α during hypoxia (red outlined upper panel) (Fig. 2B). Conversely, at doses tested, inhibition of IRE1α endoribonuclease with 4µ8c did not influence hypoxia-induced stabilization of HIF1α (Fig. 2B). We next investigated if the kinase activity of IRE1α could affect stability of HIF1α in either the cytoplasm or nucleus given its role as a transcription factor. Subcellular fractionation from hypoxic J774 monocytes-macrophages pretreated with KIRA6 confirmed that HIF1α levels are reliant on the kinase activity of IRE1α in both cytoplasmic and nuclear compartments of myeloid cells under hypoxic conditions (Fig. 2C).

Treatment with KIRA6 blunted *Hif1a* mRNA expression when compared to vehicle-treated controls as determined by RT-qPCR, suggesting that inhibition of IRE1α’s kinase activity influenced *Hif1a* transcription (Fig. 2D). Similarly, peritoneal macrophages from LysM-*Ern1*^*−/−*^ mice were unable to trigger *Hif1a* gene expression during hypoxia (Fig. 2E). We did not observe any effect of HIF1α depletion on IRE1α gene expression (*Ern1*, Fig. 2F) in hypoxic peritoneal macrophages from LysM-*Hif1a*^*−/−*^ mice. These results support a regulatory role for IRE1α on *Hif1a* transcription upon hypoxic stress.

### IRE1α/XBP1 and HIF1α crosstalk regulates the myeloid inflammatory response secondary to a hypoxic stimulus

**Fig. 3:**
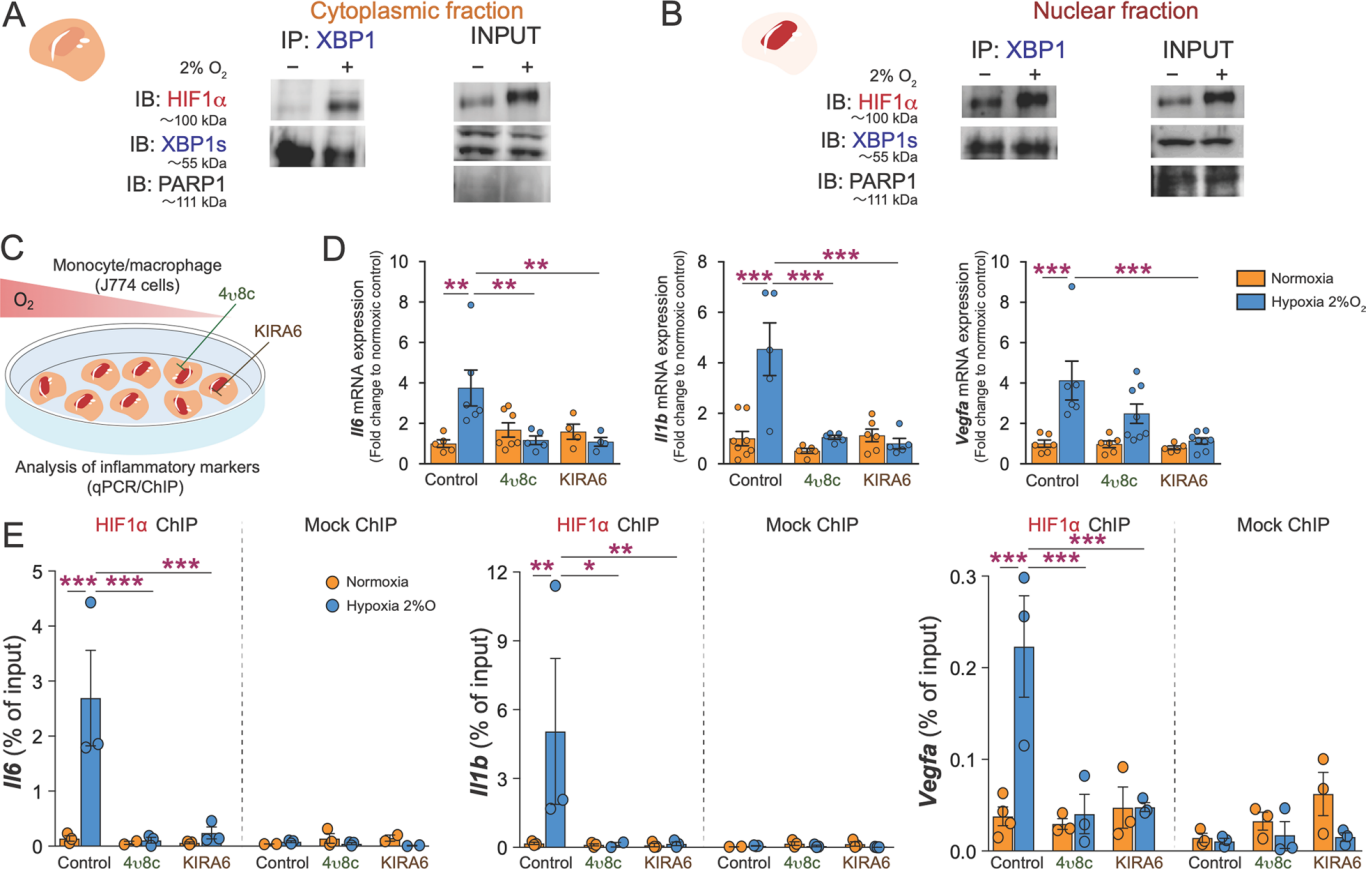
IRE1α/XBP1 and HIF1α crosstalk regulates the myeloid inflammatory response secondary to a hypoxic stimulus. **A** Co-immunoprecipitation of XBP1 and immunoblot for HIF1α in cytosolic and **B** nuclear fractions of hypoxic (2% O_2_ for 1 h) J774 cells (*n* = 2 independent experiments). **C**, **D** Schematic representation and RT-qPCR analysis of *Il6, Il1b*, and *Vegfa* mRNA expression in hypoxic (2% O_2_ for 8 h) J774 cells preincubated for 1 h with IRE1α endoribonuclease inhibitor 4µ8c (100µM) or IRE1α kinase inhibitor KIRA6 (1µM) (*n* = 3–8 per condition). **E** HIF1α or mock (IgG) ChIP-qPCR at *Il6, Il1b*, and *Vegfa* loci in hypoxic (2% O_2_ for 8 h) J774 macrophages preincubated for 1 h with IRE1α endoribonuclease inhibitor 4µ8c (100µM) or IRE1α kinase inhibitor KIRA6 (1µM) (*n* = 3 independent experiments). Percent of input represents the signals obtained from the HIF1α ChIP over signals from respective input samples. Data expressed as mean ± S.E.M. Statistical analysis (**D**, **E**): one-way ANOVA with Bonferroni post hoc analysis; *P < 0.05, **P < 0.01, and ***P < 0.001

We next set out to determine where the interplay between HIF1α and IRE1α originates. Under hypoxic conditions, HIF1α and XBP1s precipitated together in both cytoplasmic and nuclear compartments of J774 monocyte-macrophage (Fig. 3A, B) suggesting a proximal interaction. Given that IRE1α /XBP1 and HIF1α pathways have independently been described to partake in hypoxia-induced expression of pro-inflammatory genes [32, 33], we sought to assess the requirement of their interaction in a hypoxia-induced inflammatory response. Exposure of J774 monocyte-macrophages to hypoxic conditions resulted in induction of transcripts for pro-inflammatory cytokines interleukin 1 beta (*Il1b*) and interleukin 6 (*ll6*)*,* pro-angiogenic vascular endothelial growth factor A (*Vegfa*) (Fig. 3C, D) as well as tumor necrosis factor alpha (*Tnf*) (Additional file 1: 2A). Inhibition of IRE1α’s kinase signaling with KIRA6 attenuated hypoxia-driven induction of all investigated genes, while inhibition of the endoribonuclease domain with 4µ8c prevented induction of all assessed genes except *Vegfa* (Fig. 3D).

In light of IRE1α /XBP1 signaling being a candidate co-regulator of the HIF1α-induced hypoxia response, we investigated the effect of selective inhibition of IRE1α’s endoribonuclease or kinase activities on the transcription of HIF1α target genes by chromatin immunoprecipitation (ChIP)-qPCR during hypoxia (Fig. 3E). We detected increased binding of HIF1α to promoters of target genes *ll6, Il1b* and *Vegfa* in hypoxic J774 monocyte-macrophages (Fig. 3E). Blockade of either endoribonuclease (4µ8c) or kinase domains of IRE1α (KIRA6) abrogated binding of HIF1α to the promoter regions of *ll6*, *Il1b* and *Vegfa* genes during response to hypoxia (Fig. 3E). Taken together, these findings support the role of IRE1α in driving HIF1α-induced inflammatory and pro-angiogenic gene transcription in myeloid cells during hypoxia.

### Myeloid-resident HIF1α and IRE1α influence inflammation in retinal ischemia

**Fig. 4:**
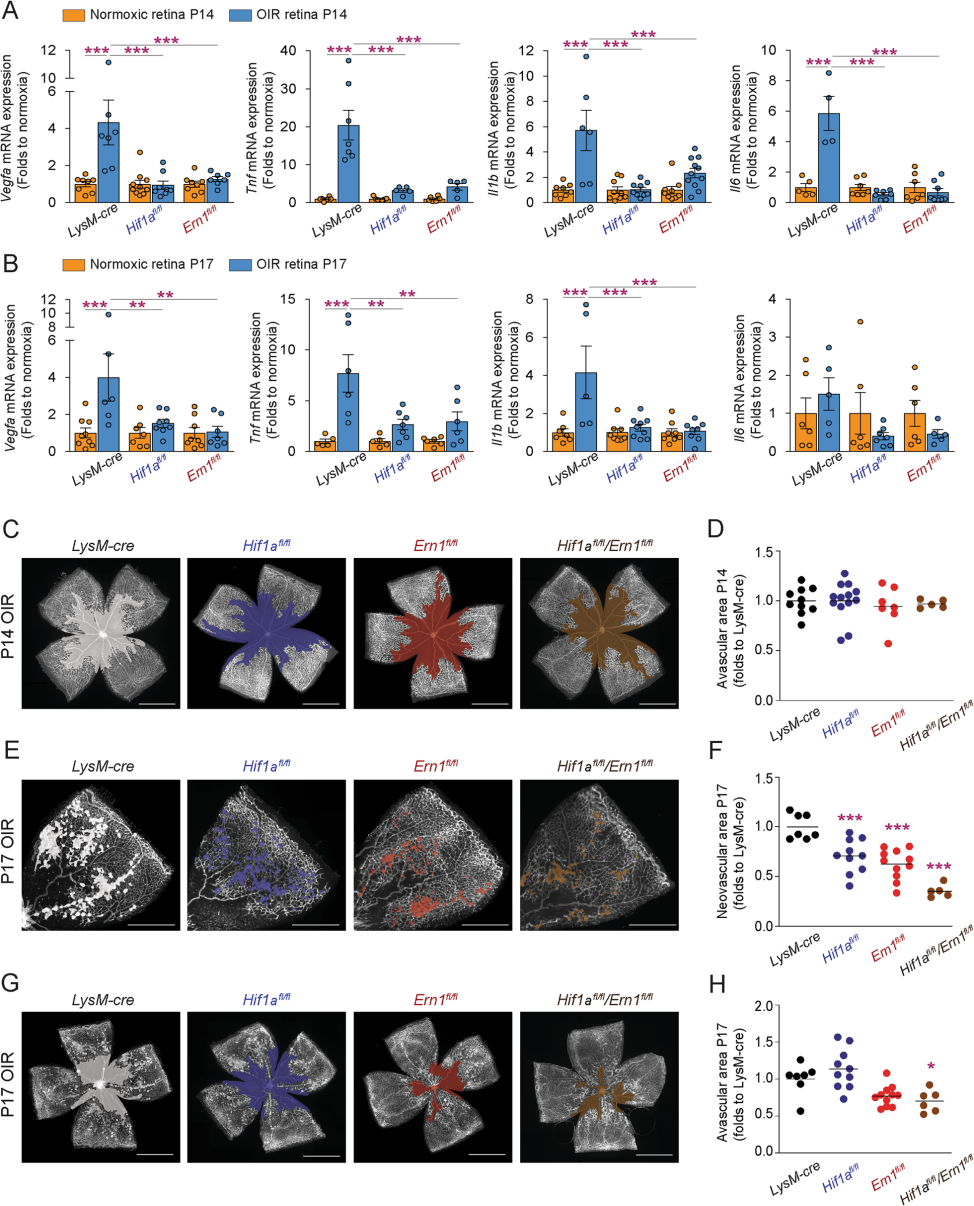
Myeloid-resident HIF1α and IRE1α influence sterile inflammation. **A** RT-qPCR analysis of *Vegfa*, *Tnf*, *Il1b*, and *Il6* mRNA expression in retinas from LysM-*Hif1a*^−/−^ and LysM-*Ern1*^−/−^ mice and their control LysM-cre*/Hif1a*^+*/*+^*/Ern1*^+*/*+^ mice conditions at P14 and **B** P17 under normoxia and OIR conditions. *n* = 5–8 retinas per condition. Results are shown as a fold change relative to respective normoxia control for each time point ± S.E.M. **C–H** LysM-*Hif1a*^−/−^, LysM-*Ern1*^−/−^, LysM-cre*/Hif1a* ^*fl/f*^*/Ern1*^*fl/fl*^ mice and their control LysM-cre*/Hif1a* ^+*/*+^*/Ern1*^+*/*+^ mice were subjected to OIR, and retinas were collected at P14 and P17, flat-mounted, and stained with isolectin B4. **C**, **D** Representative photomicrographs of isolectin B4-stained LysM-*Hif1a*^−/−^, LysM-*Ern1*^−/−^, LysM-cre*/Hif1a*^*fl/f*^*/Ern1*^*fl/fl*^ and LysM-cre*/Hif1a*^+*/*+^*/Ern1*^+*/*+^ mice at P14 with highlighted avascular hypoxic regions, and their quantification. **E**, **F** Representative photomicrographs of isolectin B4-stained LysM-*Hif1a*^−/−^, LysM-*Ern1*^−/−^, LysM-cre*/Hif1a* ^*fl/f*^*/Ern1*^*fl/fl*^ and LysM-cre*/Hif1a*^+*/*+^*/Ern1*^+*/*+^ mice at P17 with highlighted pathological neovascularization, and their quantification. **G**, **H** Representative photomicrographs of isolectin B4-stained LysM-*Hif1a*^−/−^, LysM-*Ern1*^−/−^, LysM-cre*/Hif1a* ^*fl/f*^*/Ern1*^*fl/fl*^ and LysM-cre*/Hif1a*^+*/*+^*/Ern1*^+*/*+^ mice at P17 with highlighted avascular hypoxic regions, and their quantifications. *n* = 5–13 retinas per group (**C**–**H**). Scale bars: 1 µm (for the whole flat mount of retina) and µm (for one petal of retina flat mount). Data expressed as mean ± S.E.M. Statistical analysis (**A**, **B**, **D**, **F**, **H**): one-way ANOVA with Bonferroni post hoc analysis; *P < 0.05, **P < 0.01, and ***P < 0.001

As part of the sterile inflammatory response that accompanies ischemic retinopathies, myeloid cells play a critical role in retinal neovascularization and vascular remodeling [28, 34–37]. However, this might not occur through local myeloid-mediated delivery of VEGF-A [38]. In a mouse model of OIR [22], we investigated the contribution of myeloid-resident IRE1α and HIF1α in the inflammatory response during neovascularization in mice deficient for myeloid-resident IRE1α (LysM-cre*/Ern1*^*fl/fl*^*)* and HIF1α (LysM-cre*/Hif1a*^*fl/fl*^*)*. Retinas from both mice displayed significantly less inflammatory and angiogenic cytokine transcripts such as *Il1b*, *ll6*, *Tnf *and *Vegfa* at P14 and P17 OIR (Fig. 4A, B)*. Il6* levels did not significantly vary from baseline during peak neovascularization at P17 of OIR (Fig. 4B).

We next evaluated the impact of myeloid-deficient HIF1α, IREα or both (LysM-cre*/Hif1a*^*fl/fl*^*/Ern1*^*fl/fl*^) on vascular phenotypes at P14 (during the onset of hypoxia-driven neovascularization) and P17 (at peak preretinal neovascularization). At P14, we did not observe any difference in magnitude of avascular areas suggesting that neither myeloid-resident IRE1α- nor HIF1α-mediated events were involved in hyperoxia-driven vascular degeneration (Fig. 4C, D). Importantly, during maximal neovascularization at P17, genetic deletion of myeloid *Ern1*, *Hif1a* or both significantly reduced pathological angiogenesis with LysM-cre*/Hif1a*^*fl/fl*^ mice showing a 29% reduction in neovascularization, LysM-cre*/Ern1*^*fl/fl*^ a 38% reduction and LysM-cre*/Hif1a*^*fl/f*^*/Ern1*^*fl/fl*^ a 65% reduction (Fig. 4E, F). Interestingly, LysM-cre*/Hif1a*^*fl/fl*^*/Ern1*^*fl/fl*^ showed the greatest reduction suggesting collaborative modulation of HIF1α and IRE1α signaling pathways. This is further underscored by the observation that the sole depletion of IRE1α accelerated beneficial vascular regeneration, whereas additional deletion of HIF1α further potentiated reparative angiogenesis (Fig. 4G, H). Collectively, these data highlight the role of stress response regulators HIF1α and IRE1α within myeloid cells in hypoxia-driven retinal angiogenesis.

## Discussion

The innate immune system has evolved to withstand and operate in noxious conditions. Here, we demonstrate the collaboration of 2 primitive stress response pathways in ensuring proper function of myeloid cells under hypoxic conditions. We provide evidence that under hypoxic stress, HIF1α in myeloid cells interacts through a complex with the ER-resident chaperone GRP78 and IRE1α to regulate the inflammatory response. IRE1α kinase activity influences HIF1α stabilization and potentially nuclear localization. Either IRE1α kinase activity or IRE1α endoribonuclease alone modulates HIF1α-dependent transcription of cytokines in myeloid cells. While both HIF1α and IRE1α have independently been implicated in cytokine production [14, 15, 39, 40], we provide insight on their collaboration during sterile inflammation and suggest that IRE1α is an important regulator of HIF1α activity during innate immune response of myeloid cells.

Several regulators of HIF1α activity have been identified, including chaperones such as HSP90 or HSP70, which affect HIF1α stability [41, 42]. To better understand the hypoxic response in MNPs during conditions of low oxygen, we immunoprecipitated HIF1α and performed MS/MS to identify potential binding partners. A candidate of interest was GRP78, an ER chaperone with central roles in the UPR [17–19]. In ischemic/hypoxic conditions, processes of adaptive proteostasis are triggered leading to a general reduction in translation and selective adjustment for production of proteins that are critical for survival [21, 43]. Consequently, low oxygen triggers pathways of ER stress [44]. In our hands, neither PERK nor ATF6 co-precipitated with HIF1α, suggesting selective interaction with IRE1α under hypoxic conditions. Ultimately, ChIP revealed that pharmacological inhibition of either the endoribonuclease or kinase domains of IRE1α abrogated hypoxia-driven binding of HIF1α to chromatin binding sites with the promoters of inflammatory and pro-angiogenic genes such as *Il1b*, *Il6* and *Vegf*. These findings provide additional insight on the upstream events leading to HIF1α and XBP1 collaboration in tumors under low oxygen tension [45] and suggest that kinase signaling from IRE1α, which ultimately regulates endoribonuclease activity, to be a precursory upstream event.

The mechanisms underlying HIF1α-induced hypoxia response have been extensively studied for the past three decades [8–11] and implication of HIF signaling in retinal vasculopathies [46–52] has been established. Our findings were consolidated in the OIR model of retinal ischemia-driven sterile inflammation and pathological angiogenesis where both hypoxia and myeloid cells play central roles [28, 34, 36, 37, 53, 54]. While myeloid cell-derived VEGFA may not be sufficient to cause pathological angiogenesis in OIR [38], we found that targeting IRE1α/HIF1α signaling nodes in these cells ameliorates disease outcome. Consistent with a role in driving hypoxia-induced neovascularization, we observed significant reductions in pathological preretinal neovascularization in retinas from LysM-cre*/Hif1a*^*fl/fl*^ mice. Similarly, myeloid-resident HIF1α has been implicated in vascular inflammation and angiogenesis with impacts on atherosclerosis [55], femoral arterial injury [56] and hindlimb ischemia [57]. In line with IRE1α regulating HIF1α, we observed superior reductions in pathological neovascularization when *Ern1* was knocked-out from myeloid cells (either with HIF1α or alone). Interestingly, absence of *Hif1a* alone from myeloid cells did not significantly impact beneficial vascular regeneration suggesting a selective influence on preretinal neovascularization. These data support the idea that IRE1α regulates HIF1α-driven genes that partake in pathological angiogenesis during retinopathy [45, 58].

## Conclusion

In summary, we identified a myeloid-based mechanism where IRE1α modulates the HIF1α-mediated hypoxia response. Given that current standards of care for diseases characterized by aberrant angiogenesis such as neovascular age-related macular degeneration and diabetic retinopathy often lose efficacy over time [59], therapeutic targeting of IRE1α may provide additional benefits. More fundamentally, our study identifies a node by which cellular machinery classically involved in ensuring protein quality control regulates hypoxia-driven cytokine production in myeloid cells.

## Material and methods

### Animals

All studies were performed according to the Association for Research in Vision and Ophthalmology (ARVO) Statement for the Use of Animals in Ophthalmic and Vision Research and were approved by the Animal Care Committee of the University of Montreal in agreement with the guidelines established by the Canadian Council on Animal Care. C57BL/6J, LysM-cre and *Hif1*α floxed mice were purchased from The Jackson Laboratory and CD1 nursing mothers from Charles River Laboratory. *Ern1* floxed mice were generated as in [60].

### Oxygen-induced retinopathy

Mouse pups (LysM-Cre*/Hif1a*^+*/*+^*/Ern1*^+*/*+^, LysM-cre/*Hif1a*^*fl/fl*^, LysM-cre/*Ern1*^*fl/fl*^ or LysM-cre/*Hif1a*^*fl/fl*^* /Ern1*^*fl/fl*^) and their fostering mothers (CD1, Charles River) were exposed to 75% O_2_ from postnatal day P7 to P12, then returned to room air. This model serves as a proxy to human ocular neovascular diseases such as diabetic retinopathy, which is characterized by a late phase of destructive pathological angiogenesis. Upon return to room air, hypoxia-driven neovascularization develops from P14 onward. We enucleated eyes at different time points and removed the retinas for FACS analysis or mRNA analysis. Dissected retinas were flat-mounted and incubated overnight with Fluorescein Lectin (#ZD0118, Vector Labs, 1:100) in PBS to determine the extent of avascular area or neovascularization area at P17 using ImageJ and the SWIFT-neovascularization method. Avascular areas are calculated by dividing the central capillary free area by the total retinal area. The percentage of neovascularization is calculated by dividing the area of neovascular tufts (saturated lectin-stained vasculature on the surface of the retina) by the total area of the retina.

### Cell culture and transfection studies

J774 cells were cultured in Dulbecco modified Eagle medium (DMEM) supplemented with 10% fetal bovine serum (FBS), 2.0 mM l-glutamine, 1.5 mg/mL sodium bicarbonate, 1% streptomycin/penicillin. For stimulation experiments, cells were previously starved for 5 h in the basal medium (without fetal bovine serum). Pre-treatment with 100 μM 4µ8c (#412512, EMD Millipore) or 1 μM KIRA6 (#532281, Calbiochem) was done 1 h prior to stimulation with 2% O_2_ (1 h for co-immunoprecipitation experiments and 8 h for MS/MS experiment, RNA isolation and XBP1 splicing analysis).

### FACS and cell sorting of single cell suspension from retinas

Retinas from WT mice were homogenized and incubated in a solution of 750U/mL DNase I (#69182, Sigma) and 0.5 mg/mL collagenase D (# 11088882001, Roche) for 15 min at 37 °C with gentle shaking. Homogenates were then filtered with a 70-μm cell strainer and washed in PBS, 3% FBS. Retina cell suspension was incubated with LEAF purified anti-mouse CD16/32 (# 101301, Biolegend) for 15 min at room temperature to block Fc receptors. Cells were then incubated for 30 min at room temperature with the following antibodies: FITC anti-mouse/human CD11b (# 101206, Biolegend), PE/CY7 anti-mouse Ly-6G/Ly-6C (Gr-1; #108416, Biolegend), Pacific Blue anti-mouse F4/80 (#122612, Biolegend) and 7AAD (# 559925, BD Biosciences). Microglia/macrophages cells were sorted on a BD ARIA III and processed for western blot assay.

### Primary peritoneal macrophages culture

Adult LysM-Cre/*Hif1a*^+*/*+^/*Ern1*^+*/*+^, LysM-cre/*Hif1a*^*fl/fl*^ or LysM-cre/*Ern1*^*fl/fl*^ mice (8–12 weeks old) were anesthetized with 2% isoflurane in oxygen 2 L/min and then euthanized by cervical dislocation. Then, a small incision in abdominal skin of mouse was performed. Skin was pulled to each size of the mouse, and the peritoneal cavity was washed with 5 ml PBS 3% FBS for 2 min. Then, the harvested cells were centrifuged for 5 min at 100*g*, resuspended in medium (DMEM F12 plus 10% FBS and 1% streptomycin/penicillin), and plated. After 1 h of culture at 37 °C in a humidified incubator with 5% CO_2_, the medium was changed and cells were cultured for the next 24 h in the same conditions before their hypoxic stimulation (8 h with 2%O_2_) and RT-PCR assay.

### Immunoprecipitation

For immunoprecipitations, cells were lysed in lysis buffer containing 1% NP- 40, 0.1% SDS, 0.1% deoxycholic acid, 50 mM Tris (pH 7.4), 0.1 mM EDTA, 0.1 mM EGTA, 20 mM sodium fluoride, 1 mM sodium pyrophosphate and 1 mM sodium orthovanadate. Soluble proteins were incubated with primary antibodies (2 μg) at 4 °C overnight with agitation. The following antibodies were used: Rabbit anti-HIF1α (#100479, Novus Biologicals), Rabbit anti-GRP78 (or HSPA5; #21685, Abcam) and Rabbit anti-XBP1 (#sc-7160, Santa-Cruz). 50 μL Protein A-Sepharose (#P9424, Sigma) was added and incubated for 2 h at 4 °C with agitation. The immune complexes were precipitated by centrifugation, washed 4 times with lysis buffer, boiled for 5 min in Laemmli sample buffer (#1610737, BioRad), separated by SDS-PAGE, transferred onto a nitrocellulose membrane and western blotted. Antibody detection was performed by a chemiluminescence-based detection system (ECL, #32106, Thermo Fisher scientific).

### Western blotting

J774 cells and peritoneal macrophages were cultured under hypoxia (2% O_2_) at different time points. Protein concentration from cell lysates was assessed by bicinchoninic acid assay (#BCA1, Sigma). Protein lysates were prepared in Laemmli sample buffer (#1610737, BioRad) followed by boiling at 95 °C for 5 min. The proteins were separated by SDS-PAGE and western blotting was performed by transferring proteins onto a nitrocellulose membrane. Membranes were blocked in 5% milk or 5% BSA in TBST. The primary antibodies used in this study are: anti-HIF1α (#100479, Novus Biologicals); anti-p-IRE1α^ser724^ (#48187, Abcam), anti-total IRE1α (#14C10, Cell Signaling), anti-XBP1(#sc-7160, Santa-cruz), anti-PERK (#377400, Santa-Cruz), anti-ATF6 (#166659, Santa-Cruz), and anti-ubiquitin (#sc-8017, Santa-Cruz). Secondary antibodies used in this study are: Goat Anti-Rabbit IgG (H + L)-HRP Conjugate (#1706515, BioRad) and Goat Anti-mouse IgG (H + L)-HRP Conjugate (#1706516, BioRad). HRP-conjugated blots were developed by using a chemiluminescence-based detection system (ECL, #32106, Thermo Fisher scientific).

### Preparation of samples for tandem MS/MS

J774 cells were cultured under hypoxia for 8 h. Cells lysates concentrations were assessed by bicinchoninic acid assay (#BCA1, Sigma), and then 2 mg of protein was immunoprecipitated with HIF1α antibody. The immunoprecipitate was loaded on an SDS-PAGE gel. Gel fragments were cut and sent for peptide identification by tandem mass spectrometry (MS/MS) at the IRIC proteomics center (https://capca.iric.ca/proteomics).

### Immunofluorescence

For visualization of pan-retinal vasculature, flat-mount retinas were stained with Fluorescein Lectin (#ZD0118, Vector Labs, 1:100) and observed with an epifluorescence microscope.

### Real-time quantitative PCR analysis

RNA extraction was performed with TRIzol® Reagent (#15596026, Thermo Fisher scientific) as suggested by manufacturer protocol. DNase digestion to prevent amplification of genomic DNA was then performed (#18068015, Invitrogen). 5X all in one RT mastermix (#G490, ABM) was used to generate cDNA from 1 μg of total RNA. qPCR was performed to quantify gene expression using Bright green 2X qPCR mastermix (#Mastermix-LR, ABM) and was processed with an ABI 7500 Real-Time PCR machine. *Actb* was used as a reference gene. Primers are listed in the key resources table.

### Chromatin immunoprecipitation (ChIP)

Approximately 1 million of cells were used for each ChIP experiment. Cells were fixed in 1% formaldehyde for 8 min at room temperature. 0.125 M glycine was added to stop the fixation, then cells were scraped in ice cold 1X PBS. Cells were pelleted, lysed in a Farnham lysis buffer (5 mM PIPES, 85 mM KCl, 0.5% NP-40) supplemented with 100 mM PMSF. The lysed cells were sonicated in a sonication buffer (1 mM EDTA, 10 mM Tris, 0,1% SDS supplemented with 100 mM PMSF) using a COVARIS machine until a fragment size of 150–500 bp was obtained. Sheared chromatin was immunoprecipitated with 2 μg of antibody overnight at 4 °C with rotation. The next day, magnetic beads (Magna ChIP Protein A + G Magnetic Beads; #16-663, Sigma) were added to the antibody-chromatin mixes and incubated at 4 °C with rotation for 2 h. The protein-bound magnetic beads were washed 5X with LiCl IP wash buffer and 1X with TE1x buffer. Cross-links were reversed in 120 μL of IP elution buffer (1% SDS and 0.1 M NaHCO_3_) at 65 °C overnight in a PCR cycler. DNA was purified using QIAquick PCR Purification Kit (#28106, Qiagen). qPCR was performed using Bright green 2X qPCR mastermix (#Mastermix-LR, ABM) and was processed with an ABI 7500 Real-Time PCR machine. Anti-IgG immunoprecipitation and 10% input were used as controls. Antibodies used in this study are: anti-HIF1α antibody ChIP Grade (#2185, Abcam) and rabbit IgG polyclonal isotype control ChIP grade (#171870, Abcam).

### Statistical analyses

Data are presented as mean ± SEM. GraphPad Prism (GraphPad Software, San Diego, CA; www.graphpad.com) was used to analyze the statistical significance. We used Student’s t test to compare groups of two, and one-way ANOVA with Bonferroni post hoc analysis for groups of 3 and more; data with *P* < 0.05 were considered statistically different: *P < 0.05, **P < 0.01, and ***P < 0.001.

#### Key resources table

**Table:** 

Reagent or resource	Source	Identifier
Antibodies		
Anti-HIF1α	Novus Biologicals	Cat# 100479
Anti-total IRE1α	Cell Signaling	Cat# 14C10
Anti-p-IRE1α^ser724^	Abcam	Cat# 48187
Anti-XBP1 (M-186)	Santa-cruz	Cat# sc-7160
Anti-βactine (8H10D10)	Cell Signaling	Cat# 3700
Anti-PERK (B-5)	Santa-cruz	Cat# 377400
anti-ATF6 (F-7)	Santa-cruz	Cat# 166659
Anti-GRP78 (HSPA5)	Abcam	Cat# 21685
Anti-LDH (H-10)	Santa-cruz	Cat# 133123
Anti-Ubiquitin (P4D1)	Santa-cruz	Cat# sc-8017
CD11b-FITC	Biolegend	Cat# 101206
GR-1-PE/Cy7	Biolegend	Cat# 108416
F4-80-Pacific Blue	Biolegend	Cat# 122612
7AAD	BD Biosciences	Cat# 559925
LEAF purified anti-mouse CD16/32	Biolegend	Cat# 101301
Rabbit IgG, polyclonal—Isotype Control (ChIP Grade)	Abcam	Cat# 171870
Anti-HIF1α antibody ChIP Grade	Abcam	Cat# 2185
Reagents		
4µ8c	EMD Millipore	Cat# 412512
KIRA6	Calbiochem	Cat# 532281
Fluorescein Lectin	Vector Labs	Cat# ZD0118
Trizol	Thermo Fisher scientific	Cat# 15596026
DAPI	Thermo Fisher scientific	Cat# 62248
Protein A-Sepharose® 4B	Sigma	Cat# P9424
GM-CSF	Peprotech	Cat# 315-03
DNAseI	Sigma	Cat# 69182
Invitrogen™ DNase I, Amplification Grade	Invitrogen	Cat#18068015
Collagenase D	Roche	Cat# 11088882001
Pierce™ ECL Western Blotting Substrate	Thermo Fisher scientific	Cat# 32106
Laemmli sample buffer	BioRad	Cat#1610737
Bicinchoninic Acid Kit for Protein Determination	Sigma	Cat# BCA1
Pst I restriction enzyme	New England Biolabs	Cat# R0140S
5X all in one RT mastermix	ABM	Cat#G490
Bright green 2X qPCR mastermix	ABM	Cat# MasterMix-LR
RNeasy Mini Kit	Qiagen	Cat# 74104
Dynabeads™ mRNA DIRECT™ Micro Purification Kit	Thermo Fisher scientific	Cat# 61021
Fluoromount™ Aqueous Mounting Medium	Sigma	Cat# F4680
Polyethylenimine (PEI)	Sigma	Cat#764604
Goat Anti-Rabbit IgG (H + L)-HRP Conjugate	BioRad	Cat# 1706515
Goat Anti-mouse IgG (H + L)-HRP Conjugate	BioRad	Cat# 1706516
Trypsin-EDTA Solution 1X	Sigma	Cat# 59417C
Magna ChIP Protein A + G Magnetic Beads	Sigma	Cat# 16-663
Experimental Models: Cell Lines		
J774A.1	ATCC	Cat# TIB-67
Experimental Models: Organisms/Strains		
Mouse: C57BL/6J	The Jackson Laboratory	# 00064
Mouse: B6.129P2-Lyz2tm1(cre)Ifo/J	The Jackson Laboratory	# 004781
Mouse: IRE1alpha^fl/fl^	Kind gift from R.J Kaufman	https://www.embopress.org/doi/10.1038/emboj.2011.52
Mouse: HIF1alpha^fl/fl^	The Jackson Laboratory	# 007561
Oligonucleotides for qPCR		
Mouse *Actb* Forward	This paper	5′-GAC GGC CAG GTC ATC ACT ATT G-3′
Mouse *Actb* Reverse	This paper	5′-CCA CAG GAT TCC ATA CCC AAG A-3′
Mouse *Hif1a* Forward	This paper	5'-CGAGAACGAGAAGAAAAAGATGAG-3'
Mouse *Hif1a* Reverse	This paper	5'-AAGCCATCTAGGGCTTTCAG-3'
Mouse *Ern1* Forward	This paper	5'-ATG GCA GGA TCA AGG CGA TG-3'
Mouse *Ern1* Reverse	This paper	5'-CTT CAC TCA GCA TCT CTG GGG-3'
Mouse *Il6* Forward	This paper	5′-CTT CCA TCC AGT TGC CTT C-3′
Mouse *Il6* Reverse	This paper	5′-ATT TCC ACG ATT TCC CAG AG-3′
Mouse *Il1b* Forward	This paper	5′-CTG GTA CAT CAG CAC CTC ACA-3′
Mouse *Il1b* Reverse	This paper	5′-GAG CTC CTT AAC ATG CCC TG-3′
Mouse *Vegfa* Forward	This paper	5′-GCC CTG AGT CAA GAG GAC AG-3′
Mouse *Vegfa* Reverse	This paper	5′-CTC CTA GGC CCC TCA GAA GT-3′
Mouse *Tnf* Forward	This paper	5′-CGC GAC GTG GAA CTG GCA GAA-3′
Mouse *Tnf* Reverse	This paper	5′-CTT GGT GGT TTG CTA CGA CGT GGG-3′
Mouse XBP1u Forward for PCR	This paper	5'-AAA CAG AGT AGC AGC GCA GAC TGC-3'
Mouse XBP1u Reverse for PCR	This paper	5'-TCC TTC TGG GTA GAC CTC TGG GAG-3'
Oligonucleotides for ChIP-qPCR		
Mouse *Vegfa* Forward	This paper	5′-CCTCTGTCGTCGTACGTG-3′
Mouse *Vegfa* Reverse	This paper	5′-GTACGTGCGGTGACTCT-3′
Mouse *Il6* Forward	This paper	5′-GAGGGAGTGTGTGTCTTTGTATG-3′
Mouse *Il6* Reverse	This paper	5′GAGAAAGAGAAGCTAAAGCTGATG-3′
Mouse *Il1b* Forward	This paper	5′-ATACCTGCATACTGTGTGTGCC-3′
Mouse *Il1b* Reverse	This paper	5′-AAGTCAGGATGTGCGGAACAAAG-3′
Software and Algorithms		
Prism	Graphpad	https://www.graphpad.com

## Supplementary Information

**Additional file 1: Figure S1**. Co-immunoprecipitation of HSPA5 in J774 macrophages under normoxia and hypoxia for 1 h followed by immunoblotting for GRP78, HIF1α, and UPR sensors IRE1α, PERK and ATF6. **Figure S2. ** RT-qPCR of *Tnf* in hypoxic J774 macrophages preincubated for 1h with IRE1α endoribonuclease inhibitor 4μ8c or IRE1α kinase inhibitor KIRA6. Data expressed as mean ± S.E.M. Statistical analysis: one-way ANOVA with Bonferroni post hoc analysis; *P < 0.05.

**Additional file 2: Table S1.** Gene Set Variation analysis of normoxic and hypoxic retinal samples at P14.

**Additional file 3: Table S2. **GSVA analysis of normoxic and hypoxic retinal samples at p17.

**Additional file 4: Table S3. **List of the proteins obtained after tandem mass spectrometry analysis of immunoprecipitation of HIF1α from J774 macrophages under hypoxia.

## Data Availability

The data sets analyzed during the current study are available from the corresponding author on reasonable request.

## Abbreviations

HIF1α: Hypoxia-Inducible Factor 1α
IRE1α: Inositol-requiring enzyme 1α
GRP78: Glucose-Regulated Protein-78
UPR: Unfolded protein response
PERK: Protein kinase RNA-like ER kinase
ATF4: Activating transcription factor 4
XBP1: X-box binding protein-1
ATF6: Activating transcription factor 6
OIR: Oxygen-induced retinopathy
P: Postnatal day
GSVA: Gene set variation analysis
MNP: Mononuclear phagocyte
*Il1b*: Interleukin 1 beta (transcript)
*Il6*: Interleukin 6 (transcript)
*Tnf*: Tumor necrosis factor alpha (transcript)
*Vegfa*: Vascular endothelial growth factor A (transcript)
ChIP: Chromatin Immunoprecipitation
HSP90 (or 70): Heat Shock Protein 90 (or 70)
