# Supplementary material for: HIF1α-dependent hypoxia response in myeloid cells requires IRE1α
Source: J Neuroinflammation. 2023 Jun 21;20:145. doi: 10.1186/s12974-023-02793-y (PMC10286485; doi:10.1186/s12974-023-02793-y)
Supplement: Supplementary file 1 — Additional file 1: Figure S1. Co-immunoprecipitation of HSPA5 in J774 macrophages under normoxia and hypoxia for 1 h followed by immunoblotting for GRP78, HIF1α, and UPR sensors IRE1α, PERK and ATF6. Figure S2. RT-qPCR of Tnf in hypoxic J774 macrophages preincubated for 1h with IRE1α endoribonuclease inhibitor 4μ8c or IRE1α kinase inhibitor KIRA6. Data expressed as mean ± S.E.M. Statistical analysis: one-way ANOVA with Bonferroni post hoc analysis; *P < 0.05. [file 12974_2023_2793_MOESM1_ESM.pdf]

SUPPLEMENTAL FIGURE 1

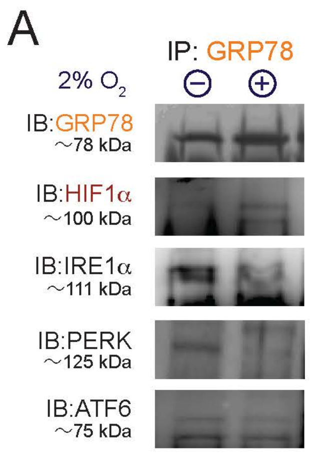

SUPPLEMENTAL FIGURE 2

A

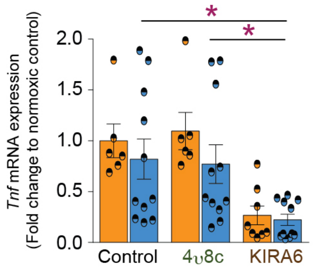

SUPPLEMENTAL FIGURE LEGENDS

Supplementary Table 1. Gene Set Variation Analysis (GSVA) analysis of normoxic and hypoxic retinal samples at P14.

Supplementary Table 2. GSVA analysis of normoxic and hypoxic retinal samples at p17.

Supplementary Table 3. List of the proteins obtained after tandem mass spectrometry (MS/MS) analysis of immunoprecipitation of HIF1α from J774 macrophages under hypoxia (2% O_2 for 8h).

Supplementary Figure 1. (A) Co-immunoprecipitation of HSPA5 in J774 macrophages under normoxia (21% O_2) and hypoxia (2% O_2) for 1h followed by immunoblotting (IB) for GRP78, HIF1α, and UPR sensors IRE1α, PERK and ATF6 (n=3 independent experiments).

Supplementary Figure 2.

(A) ChIP-qPCR of *Tnf* in hypoxic (2% O_2 for 8h) J774 macrophages preincubated for 1h with IRE1α endoribonuclease inhibitor 4μ8c (100μM) or IRE1α kinase inhibitor KIRA6 (1μM) (n= 3 independent experiments). ChIP assay was performed using anti-HIF1α polyclonal antibody for the detection of enriched DNA fragments. Percent of input represents the signals obtained from the HIF1α ChIP over signals from respective input samples. Data expressed as mean ± S.E.M. Statistical analysis: one-way ANOVA with Bonferroni post-hoc analysis; $*P < 0.05$.
